# Ultrasound imaging of the anterior section of the eye of five different snake species

**DOI:** 10.1186/s12917-014-0313-5

**Published:** 2014-12-30

**Authors:** Henrik Lauridsen, Mari-Ann O Da Silva, Kasper Hansen, Heidi M Jensen, Mads Warming, Tobias Wang, Michael Pedersen

**Affiliations:** Department of Clinical Medicine, Comparative Medicine Lab, Aarhus University, Brendstrupgaardsvej, Aarhus N, Denmark; Center for Zoo and Wild Animal Health, Copenhagen Zoo, Roskildevej, Frederiksberg, Denmark; Eye Pathology Institute, University of Copenhagen, Frederik V’s Vej, Copenhagen, Denmark; Department of Biosciences, Zoophysiology, Aarhus University, DK-8000 Aarhus, Denmark

**Keywords:** Snake, Spectacle, Ultrasound

## Abstract

**Background:**

Nineteen clinically normal snakes: six ball pythons (*Python regius*), six Burmese pythons (*Python bivittatus*), one Children’s python (*Antaresia childreni*), four Amazon tree boas (*Corallus hortulanus*), and two Malagasy ground boas (*Acrantophis madagascariensis*) were subjected to ultrasound imaging with 21 MHz (ball python) and 50 MHz (ball python, Burmese python, Children’s python, Amazon tree boa, Malagasy ground boa) transducers in order to measure the different structures of the anterior segment in clinically normal snake eyes with the aim to review baseline values for clinically important ophthalmic structures. The ultrasonographic measurements included horizontal spectacle diameter, spectacle thickness, depth of sub-spectacular space and corneal thickness. For comparative purposes, a formalin-fixed head of a Burmese python was subjected to micro computed tomography.

**Results:**

In all snakes, the spectacle was thinner than the cornea. There was significant difference in spectacle diameter, and spectacle and corneal thickness between the Amazon tree boa and the Burmese and ball pythons. There was no difference in the depth of the sub-spectacular space. The results obtained in the Burmese python with the 50 MHz transducer were similar to the results obtained with micro computed tomography. Images acquired with the 21 MHz transducer included artifacts which may be misinterpreted as ocular structures.

**Conclusions:**

Our measurements of the structures in the anterior segment of the eye can serve as orientative values for snakes examined for ocular diseases. In addition, we demonstrated that using a high frequency transducer minimizes the risk of misinterpreting artifacts as ocular structures.

**Electronic supplementary material:**

The online version of this article (doi:10.1186/s12917-014-0313-5) contains supplementary material, which is available to authorized users.

## Background

**Figure 1:**
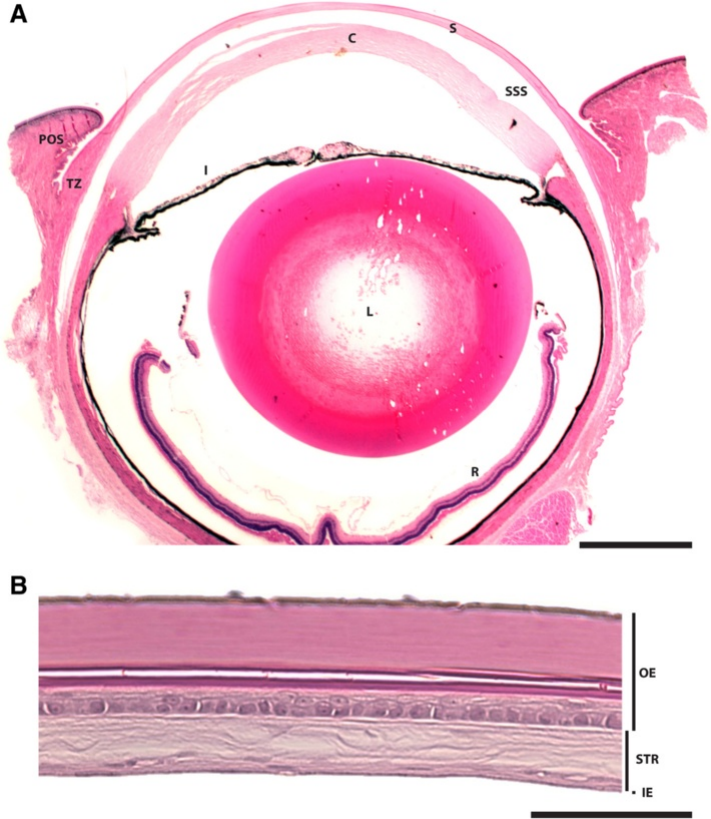
**Histological images of the eye of a ball python (** ***Python regius*** **).** Transversal histological section through the medial portion of the whole eye **(A)** and spectacle **(B)** stained with hematoxylin and eosin. Cornea (C), inner epithelium (IE), iris (I), lens (L), outer epithelium (OE), periocular scale (POS), retina (R), spectacle (S), stroma (STR), sub-spectacular space (SSS), and spectacular transition zone (TZ). Scale bar in **(A)** is 1 mm and 100 μm in **(B)**. Histological image in **(A)** is from [7] and reprinted with permission from John Wiley and Sons.

The snake eye differs considerably from that of mammals, with the most striking difference being the absence of moveable eyelids. The eyelids in snakes fuse during the embryological development [1-4] and, in contrast to mammalian eyelids, they do not reopen and become transparent forming the spectacle [1,2,5,6]. Histological evaluation shows that the spectacle resembles the cornea, but it is thinner and contains nerves and blood vessels [7]. The spectacle consists of three layers (Figure 1); an outer epithelium with basal cells and overlying keratin; a central stroma consisting of organized collagen fibrils; and an inner epithelium with flat cells with microvilli and fluid filled vesicles [7]. The keratin layers of the outer epithelium participate in the periodic shedding of the snake skin, making this layer dynamic during the renewal phases [8].

Between the spectacle and the cornea a narrow sub-spectacular space is found [4]. This space receives fluid from a large post-ocular Harderian gland and drains through a lacrimal system into the roof of the mouth [3]. This drainage system may be the port of entry for ocular pathogens coming from the respiratory tract or the mouth.

High-frequency ultrasound transducers (>30 MHz) provide excellent images of the eye and have become useful for diagnosing ocular diseases. Nevertheless, reference values of ocular dimensions in healthy animals are pivotal to fully exploit the enormous clinical potential of ultrasonography.

The eyes of nineteen clinically normal snakes were ultrasonographically examined by using low and high frequency transducers. The measurements obtained from different species were compared. For comparison of measurements and thereby evaluation of the examination method, a formalin-fixed head of a Burmese python was subjected to X-ray micro computed tomography (μCT). A critical evaluation of the ultrasound method is made and morphometric baseline data of spectacle and cornea of five species of snakes are presented.

## Methods

### UItrasound imaging

Nineteen snakes from the family Pythonidae and family Boidae were examined. All snakes were considered healthy with no history of disease. The five examined species were ball python (*Python regius*) (n = 6), Burmese python (*Python bivittatus*) (n = 6), Children’s python (*Antaresia childreni*) (n = 1), Amazon tree boa (*Corallus hortulanus*) (n = 4), and Malagasy ground boa (*Acrantophis madagascarensis*) (n = 2). Snakes of each species were similar in length. Gender was not recorded. The examinations were approved by the Danish Animal Experiments Inspectorate.

Before ultrasound examination, each snake was anaesthetized by saturating the snake’s container with the inhalant anesthetic sevoflurane (Sevofluran, Baxter) by introducing a sevoflurane-impregnated cotton wool. The snakes were intubated and ventilation with room air was manually maintained by using a neonatal resuscitator. Body length and weight were recorded (Table 1). Ultrasound was carried out using a high frequency system (VisualSonics Vevo 2100, Fujifilm VisualSonics, Inc.). The snake head was fixed in a setting of soft towels, ultrasound gel was applied directly to the eye, and the transducer was mounted in an integrated rail system thereby minimizing movements during image acquisition. Both eyes of each individual snake were examined, and the mean of three separate measurements of some ocular structures: horizontal spectacle diameter, spectacle thickness, cornea thickness, and sub-spectacular depth were calculated. To assess the difference between the performance of ultrasound transducers emitting sound at different frequencies, all examinations on ball pythons were repeated using a high frequency transducer (mean frequency = 50 MHz), and a low frequency transducer (mean frequency = 21 MHz). Ultrasound examinations on all other species were performed using only the high frequency (50 MHz) transducer.

**Table 1:** **Physical, ultrasonographic (US) and micro-CT (μCT) measurements of** **five species of snakes**

**Species**	**Ball python**	**Burmese python**		**Children’s python**	**Amazon tree boa**	**Malagasy ground boa**
**Parameter**	**(US)**	**(US)**	**(μCT)**	**(US)**	**(US)**	**(US)**
n =	6	6	1	1	4	2
Body length (cm)	114.8 ± 6.0 CI95 (110.0;119.5)	102.9 ± 3.8 CI95 (99.9;105.9)	100.3	90.5	54.6 ± 18.8 CI95 (39.5;69.6)	80.5 ± 5.0 CI95 (76.5;84.5)
Body weight (g)	1309.5 ± 196.4 CI95 (1152.4;1466.7)	513.0 ± 58.1 CI95 (466.5;559.6)	491.6	318.8	45.9 ± 11.4 CI95 (36.8;55.1)	449.2 ± 57.8 CI95 (403.0;495.5)
Spectacle diameter (mm)	5.8 ± 0.5 CI95 (5.4;6.2)	5.1 ± 0.2 CI95 (4.9;5.3)	5.1	3.4	3.8 ± 0.3 CI95 (3.6;4.0)	4.9 ± 0.3 CI95 (4.7;5.1)
Central spectacle thickness (μm)	111.3 ± 3.4 CI95 (108.6;114.1)	110.1 ± 9.3 CI95 (102.6;117.5)	114.4	95.0	76.6 ± 7.9 CI95 (70.3;82.9)	94.5 ± 1.4 CI95 (93.4;95.6)
Depth of sub-spectacular space (μm)	57.3 ± 30.7 CI95 (32.8;81.9)	49.7 ± 26.2 CI95 (28.7;70.6)	65.2	26.5	23.4 ± 10.3 CI95 (15.1;31.6)	19.5 ± 5.7 CI95 (15.0;24.0)
Central corneal thickness (μm)	228.4 ± 22.2 CI95 (210.7;246.1)	237.3 ± 16.9 CI95 (223.7;250.8)	224.1	194.5	186.1 ± 3.0 CI95 (183.8;188.5)	210.5 ± 1.4 CI95 (209.4;211.6)

Values are mean ± standard deviation and 95% confidence interval (CI95).

### Micro Computed Tomography (μCT)

To compare measurements obtained by ultrasound examinations with an imaging modality that allows for acquisition of three-dimensional data of spatial structures, we applied an iodine based soft-tissue staining protocol described by Da Silva et al. [9] and performed μCT (Scanco Medical, Brüttisellen) (21.04 × 23.56 mm^2^ field-of-view; 1403 × 1571 matrix; 0.015 mm slice thickness; 55 kVp tube voltage; 116 μA tube current; 24 μm pixel pitch, resulting in a 15 μm^3^ image resolution) on a single formalin-fixed Burmese python's head.

Data were analyzed using statistical software (JMP® 9.0.2, SAS Institute, Inc.). Measurements were compared using ANOVA statistics and *p* < 0.05 was accepted as significant. The numbers of examined Children’s python (n = 1) and Malagasy ground boa (n = 2) were small, therefore interspecies comparisons were restricted to include the ball and Burmese pythons and the Amazon tree boas.

## Results

**Figure 2:**
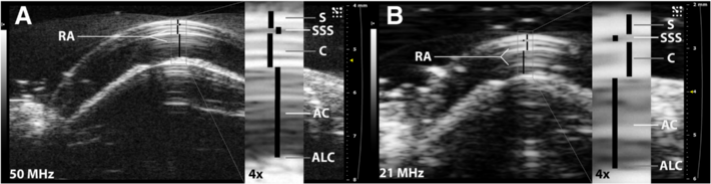
**Ultrasonographic images of the eye of a ball python (** ***Python regius*** **).** Images acquired with a 50 MHz **(A)** and a 21 MHz **(B)** transducer. Ophthalmic structures of the anterior segment are revealed at both frequencies: anterior chamber (AC), anterior lens capsule (ALC), cornea (C), spectacle (S), and sub-spectacular space (SSS). Reverberation artifacts (RA) are present at both frequencies; however they are more easily recognizable as artifacts at 50 MHz.

Ultrasound examination revealed the anatomical structures within the anterior portion of the snake eye, with a superior image quality provided by a 50 MHz transducer compared to a 21 MHz transducer (Figure 2). All morphometric measurements are listed in Table 1. The ball pythons were the overall longest and heaviest snakes, and the Amazon tree boas were the shortest and lightest individuals. There was no significant difference in the ocular measurements of the left and the right eyes, and therefore, these values were pooled for analysis.

Spectacle diameter differed significantly among all three species compared (ball python, Burmese python, and Amazon tree boa), with the ball python having the largest horizontal diameter and the Amazon tree boa the smallest (Table 1). Absolute spectacle and corneal thickness differed significantly between the boas and the pythons in this study. In all the studied snakes, the spectacle was thinner than the cornea, and there was no significant intraspecific difference in the depth of the sub-spectacular space, which varied considerably between species.

The ratio between the ocular measurements and snake body length was significant only for corneal thickness. The highest ratio was observed in the Amazon tree boa, whereas there was no significant difference between the pythons. The ratio between the four ocular measurements and the body weight showed significantly higher ratios for the Amazon tree boa and no difference between the pythons. Of the four ocular measurements, there was significant correlation only between body length and spectacle thickness.

No adverse effects were detected in any snake as a result of the ultrasound examinations.

**Figure 3:**
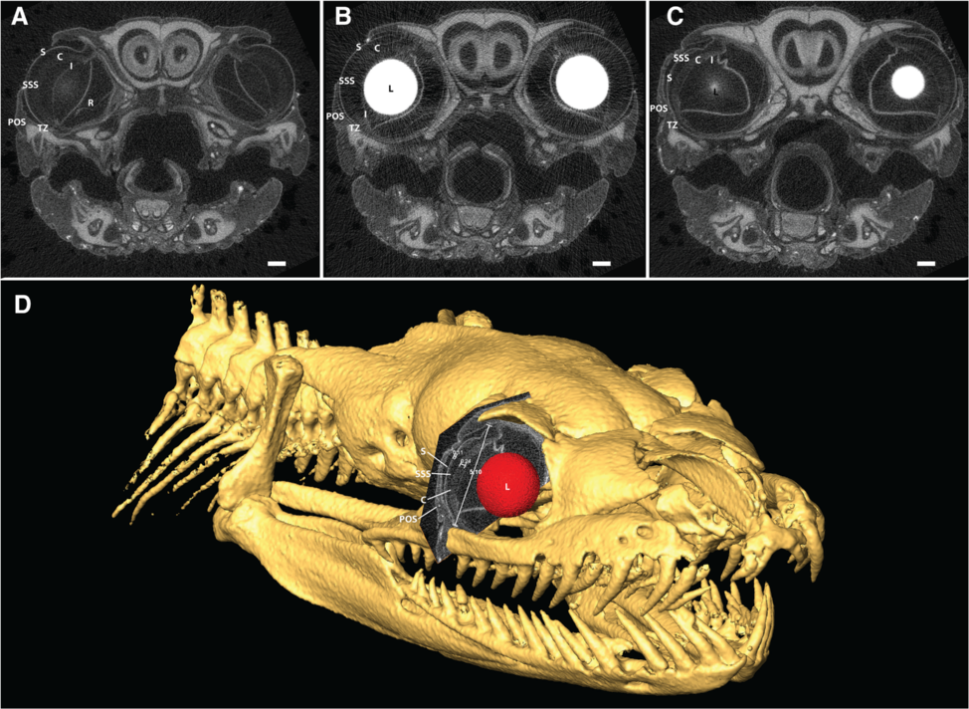
**μCT images of iodine stained Burmese python (** ***Python bivittatus*** **).** Transversal sections through the anterior **(A)**, medial **(B)**, and posterior **(C)** portions of the eye (scale bar = 1 mm). The ophthalmic structures described by ultrasound are clearly revealed at both the anterior **(A)** and posterior **(C)** sections, whereas the iodine stained lens all but shadows these structures in the medial section **(B)**. Cornea (C), iris (I), lens (L), periocular scale (POS), retina (R), spectacle (S), sub-spectacular space (SSS), and spectacular transition zone (TZ). μCT allows for three-dimensional reconstructions of spatial structures **(D)**, aiding interpretation of ultrasonographic images obtained in two dimensions.

To verify the two-dimensional description of the three-dimensional structure of the snake eyes, a single Burmese python specimen was stained with iodine for high-resolution μCT imaging (Figure 3). Ophthalmic measurements from this specimen are included in Table 1.

Blood flow in the spectacle could be observed with the high frequency ultrasound transducer in all species (Additional file 1). Additionally, we observed a distinct capillary blood flow in vessels lining the iris, most evident in the Burmese python (Additional file 1).

## Discussion

This study provides morphometric baseline values for four clinically important ophthalmic structures in five species of snakes. We found a significant difference in the spectacle diameter and corneal and spectacle thickness between pythons and boas. Furthermore, all spectacles were thinner than the corneas. There was, however, no significant difference in the depth of the sub-spectacular space. Hollingsworth et al. [10] previously reported no difference in spectacle thickness, corneal thickness and subspectacular space depth between ball pythons and California king snakes (Colubridae). They also reported that the spectacle was thicker than the cornea in the four species examined. As pythons and boas are closer related than pythons and colubrids, we anticipated no difference in the four ocular measurements performed in this study.

However, the results of our study addressed the importance of applying ultrasound at an adequate frequency when performing ophthalmic measurements of thin acoustically reflective structures. Figure 2 illustrates the difference in image quality between a 50 MHz and 21 MHz ultrasound transducer, respectively, on the same eye. Use of higher frequency generally provides higher image resolution, facilitating improved structural information about the anterior eye structures. Hollingsworth et al. [10] report the use of a 50 MHz transducer to acquire images, but present an image of comparable resolution/detail to the presented 21 MHz image of this study (Figure 2B). A variety of image artifacts have been associated with ultrasound examinations [11]. Image artifacts of particular interest when examining snake eyes are reverberations resulting from ultrasound echoes being repeatedly reflected between two highly reflective surfaces and expressed as repetitive hyperechoic lines. The anterior and posterior surfaces of the lens in human patients create reverberation artifacts [12]. We speculate that a similar phenomenon occurs when performing ultrasonography on the snake spectacle, resulting in repetitive lines emanating from the posterior surface of the spectacle down into the sub-spectacular space (Figure 2). If these lines are not recognized as image artifacts by their on/off appearance when the transducer is gently moved over the eye during image acquisition, they can easily be interpreted incorrectly as the anterior or posterior surface of the cornea. Applying a high frequency ultrasound transducer reduces the risk of such incorrect interpretations, as even minute structures are more easily recognized due to higher image resolution and quality (compare Figure 2A and 2B).

The measurements obtained in the present study may be compared to a recent study [7], wherein the eyes of ball pythons were measured using Optical Coherence Tomography (OCT). Da Silva et al. [7] clearly showed that the spectacle is thinner than the cornea and that the central spectacle thickness was 108.2 ± 13.4 μm for the ball python which is very similar to the measured 111.3 ± 3.4 μm in the present study. Additionally, μCT performed in this study allows for three-dimensional representations of minute anatomical structures [13] and revealed similar structures as described by ultrasound imaging, and ophthalmic measurements were not significantly different.. The slight differences between measurements may be attributed to formalin fixation [14].

Further studies into the blood flow in the spectacle and the vessels lining the iris make interesting areas for future research. One study [15] has shown that the vessels of the spectacle undergo cycles of dilation and constriction, the duration of which depend on the physiological state of the snake. Examining the blood flow in additional snakes species as well as in snakes with disease would provide information that could ultimately lead to improved treatment of ailing spectacles.

## Conclusions

In conclusion, we provide a series of baseline ophthalmic measurements of five species of healthy snakes, which relate well to measurements obtained by μCT and OCT. Furthermore, we advocate for the use of adequate ultrasound equipment when examining the small sound reflective structures of the eye.

## Additional file

Additional file 1:
**Ultrasonographic video of the eye of a Burmese python.** Blood flow can be clearly observed in the corneal vessels (at two o’clock in the eye globe) and also in the vessels lining the iris.

## Abbreviations

AC: Anterior chamber
ALC: Anterior lens capsule
C: Cornea
I: Iris
IE: Inner epithelium
L: Lens
OCT: Optical Coherence Tomography
OE: Outer epithelium
POS: Periocular scale
R: Retina
RA: Reverberation artifacts
S: Spectacle
SSS: Sub-spectacular space
STR: Stroma
TZ: Spectacular transition zone
μCT: Micro x-ray computed tomography
